# A population pharmacokinetic model of remdesivir and its major metabolites based on published mean values from healthy subjects

**DOI:** 10.1007/s00210-022-02292-6

**Published:** 2022-09-20

**Authors:** Ahmed Abouellil, Muhammad Bilal, Max Taubert, Uwe Fuhr

**Affiliations:** 1 Faculty of Medicine, Center for Pharmacology, Department I of Pharmacology, University Hospital Cologne, University of Cologne, Gleueler Straße 24, 50931 Cologne, Germany; 2 Immunosensation Cluster of Excellence, University Hospital Bonn, Bonn, Germany; 3 Department of Clinical Pharmacy, Institute of Pharmacy, University of Bonn, Bonn, Germany

**Keywords:** Anti-virals, Population pharmacokinetics, COVID-19, Remdesivir, Pharmacometrics, GS-441524, GS-704277

## Abstract

Remdesivir is a direct-acting anti-viral agent. It was originally evaluated against filoviruses. However, during the COVID-19 pandemic, it was investigated due to its anti-viral activities against (SARS-CoV-2) virus. Therefore remdesivir received conditional approval for treatment of patients with severe coronavirus disease. Yet, its pharmacokinetic properties are inadequately understood. This report describes the population pharmacokinetics of remdesivir and its two plasma-detectable metabolites (GS-704277 and GS-441524) in healthy volunteers. The data was extracted from published phase I single escalating and multiple i.v remdesivir dose studies conducted by the manufacturer. The model was developed by standard methods using non-linear mixed effect modeling. Also, a series of simulations were carried out to test suggested clinical doses. The model describes the distribution of remdesivir and each of its metabolites by respective two compartments with sequential metabolism between moieties, and elimination from central compartments. As individual data were not available, only inter-cohort variability could be assessed. The estimated point estimates for central (and peripheral) volumes of distribution for remdesivir, GS-704277, and GS-441524 were 4.89 L (46.5 L), 96.4 L (8.64 L), and 26.2 L (66.2 L), respectively. The estimated elimination clearances of remdesivir, GS704277, and GS-441524 reached 18.1 L/h, 36.9 L/h, and 4.74 L/h, respectively. The developed model described the data well. Simulations of clinically approved doses showed that GS-441524 concentrations in plasma exceeded the reported EC_50_ values during the complete duration of treatment. Nonetheless, further studies are needed to explore the pharmacokinetics of remdesivir and its relationship to clinical efficacy, and the present model may serve as a useful starting point for additional evaluations.

**Supplementary Information:**

The online version contains supplementary material available at 10.1007/s00210-022-02292-6.

## Introduction

Current management of patients with severe COVID-19 mainly focuses on immune response modulation and symptomatic therapy. In critically ill patients, management would also include supplemental oxygen and mechanical ventilation, along with the suppression of inadequate immune response. Healthcare providers also attempt to control secondary infections and thrombosis by either prophylaxis or treatment (Sieswerda et al. 2021; Godino et al. 2021). So far, only two anti-viral small-molecule medications have been authorized for the treatment of COVID-19 in 2020/2021, which includes the use of remdesivir under certain conditions (CDC 2021; EMA 2021; Diaz et al. 2021), and molnupiravir which has just been authorized in Great Britain for the use in patients with mild to moderate COVID-19 with risk factors for developing severe illness (UK-MHRA 2021). And recently, the FDA has expanded the use of remdesivir to certain non-hospitalized adults and pediatric patients for the treatment of mild-to-moderate COVID-19 disease (FDA 2022).

Remdesivir was originally evaluated as a broad-spectrum filovirus inhibitor that can protect against the development of Ebola virus disease (de Wit et al. 2020). During the COVID-19 pandemic, remdesivir showed anti-SARS-CoV-2 activities in vitro and in animal models. These activities were attributed to its triphosphate nucleoside analog, which acts as an inhibitor of the viral RNA-dependent RNA polymerase. However, evidence that supports its efficacy is still under investigation (Wang et al. 2020; de Wit et al. 2020; Alsayed et al. 2021). Remdesivir is also being investigated as a part of drug cocktails that might be used to treat hospitalized COVID-19 patients (Kalil et al. 2021; Vitiello and Ferrara 2021).

Remdesivir needs to go through an extensive metabolic process to be active, and from those metabolism products, only GS‐704277 and GS‐441524 can be quantified in plasma (Figure S1) (Humeniuk et al. 2021a).

To date, publicly available information on the pharmacokinetics of remdesivir and its metabolites is limited. A non-compartmental description of data in healthy volunteers has been published and serves as the basis for the present evaluation (Humeniuk et al. 2020). Several bottom-up approaches using physiologically based pharmacokinetic models have recently been reported (Deb and Reeves 2021; Fan et al. 2021; Humeniuk et al. 2021b; Gallo 2021), of which the most recent one was generated by scientists of the U.S. Food and Drug Administration (FDA) (Fan et al. 2021). Such models are very useful but in part are based on assumptions that remain to be verified, and occasionally use “optimization” of some of their predefined parameters, with no other reason than that simulations should match the observed data. As an empirical compartmental approach, the manufacturers of remdesivir developed a population pharmacokinetic model of which only parts are publicly available (LHartman et al. 2020). Unfortunately, the information provided there is not sufficient to retrace and assess the performance of the model in detail. Finally, a population pharmacokinetic model has been reported for the GS-441524 metabolite only in Japanese patients with renal impairment (Sukeishi et al. 2021). Overall, the available information reflects only a first step to support more precise dosing strategies for remdesivir. Such information is needed to integrate intrinsic and extrinsic factors for a better understanding of the pharmacokinetics and dynamics of remdesivir. The main objective of the present report was therefore to develop an independent compartmental population pharmacokinetic model that can fit the observed data obtained from literature and empirically describe the pharmacokinetic parameters of remdesivir, GS‐704277, and GS‐441524. This model may be further used in determining suitable dosing strategies in patients, with a perspective to be expanded for patients with chronic conditions.

## Methods

Arithmetic-concentration data were obtained from published randomized, blinded, placebo‐controlled, phase I program that evaluated the safety and pharmacokinetics of single and multiple ascending intravenous doses of remdesivir (Humeniuk et al. 2020). Data points were extracted using GetData Graph Digitizer software (getdata-graph-digitizer.com) and R.

In this trial, remdesivir was administrated as a single 2-h intravenous infusion at doses of 3 mg, 10 mg, 30 mg, 75 mg, 150 mg, and 225 mg. Or as a once-daily 1-h intravenous infusion for 7 and 14 days. This program was carried out in healthy male and non-pregnant, non-lactating female volunteers with an age range of 18 to 55 years and a body mass index of 18 to 30 kg/m^2^. No detailed information regarding individual parameters for each cohort was given, and the study was conducted by the manufacturing company, Gilead Sciences, Inc., USA (Humeniuk et al. 2020).

Population pharmacokinetic (PK) parameters were estimated by standard methods using non-linear mixed effect modeling software (Monolix  2019R2 - Antony, France) .

The model was developed by testing different distribution patterns and different compartment numbers for remdesivir, GS‐774277, and GS‐441524, in addition to different elimination and metabolic models that describe the conversion of remdesivir to GS‐704277, and GS‐704277 to GS‐441524. For this purpose, metabolism was assumed to occur solely in the central compartment or in the central and peripheral compartments simultaneously.

Estimated PK parameters included total body clearance (*CL*), the volume of distribution for central and peripheral compartments (*Vd*_*c*_ and *Vd*_*p*_), inter-compartmental clearance (*Q*), and formation clearance of metabolites (*CLm*). Terminal elimination half-life using regression (*t*_½ cc_) was also calculated using GraphPad Prism 8 (GraphPad Softwares, 2019, CA, USA). Microsoft Excel (Microsoft, 2021, Redmond, USA) and RStudio (PBC, 2021, Boston, USA) were used for dataset construction, analysis, and graph generation.

The published concentration–time profile of remdesivir showed a sharp increase in plasma concentration immediately following the end of the 2-h intravenous infusion. This could be explained by intravenous line saline washing following the 2-h infusion (Rita Humeniuk, personal communication). To incorporate this sudden increase of remdesivir concentrations into the model, 4% of the total administered dose was subtracted from the continuous infusion dose and given instantaneously at the end of the infusion. The choice of this method and the selected percentage were based on other published methodology (Anh et al. 2006).

The final model was chosen based on goodness-of-fit plots, visual predictive checks, metabolic plausibility, parameter shrinkage, and the − 2 × log of likelihood (Figure S2).

The most suitable model was developed using ordinary differential equations (ODE) in non-linear mixed-effect modeling software (Table 1). The probable post hoc values of each dose’s fixed parameters were calculated using empirical Bayes method in Monolix software.

**Table 1:** Ordinary differential equations that best describe the pharmacokinetic model for remdesivir and its two metabolites GS‐774277 and GS‐441524

*C*_*c* RDV_ = *A*_(RDV Central compartment)_/*Vd*_*c* RDV_
*C*_*p* RDV_ = *A*_(RDV Peripheral compartment)_/*Vd*_*p* RDV_
*C*_*c* GS‐774277_ = *A*_(GS‐774277 Central compartment)_/*Vd*_*c* GS‐774277_
*C*_*p* GS‐774277_ = *A*_(GS‐774277 Peripheral compartment)_/*Vd*_*p* GS‐774277_
*C*_*c* GS‐441524_ = *A*_(GS‐441524 Central compartment)_/*Vd*_*c* G GS‐441524_
*C*_*p* GS‐441524_ = *A*_(GS‐441524 Peripheral compartment)_/*Vd*_*p* GS‐441524_
$\frac{DA}{DT}$_(RDV Central compartment)_ = (Input) + (*Q*_RDV_ × *C*_*p* RDV_) − (*Q*_RDV_ × *C*_*c* RDV_) − (*CL*_RDV_ × *C*_*c* RDV_) − (*CLm*_*c* GS‐774277_ × *C*_*c* RDV_)
$\frac{DA}{DT}$_(RDV Peripheral compartment)_ = (*Q*_RDV_ × *C*_*c* RDV_) − (*Q*_RDV_ × *C*_*p* RDV_) − (*CLm*_*p* GS‐774277_ × *C*_*p* RDV_)
$\frac{DA}{DT}$_(GS‐774277 Central compartment)_ = (*CLm*_*c* GS‐774277_ × *C*_*c* RDV_) + (*Q*_GS‐774277_ × *C*_*p* GS‐774277_) − (*Q*_GS‐774277_ × *C*_*c* GS‐774277_) − *CL*_GS‐774277_ × *C*_*c* GS‐774277_) − (*CLm*_*c* GS‐441524_ × *C*_*c* GS‐774277_)
$\frac{DA}{DT}$_(GS‐774277 Peripheral compartment)_ = (*Q*_GS‐774277_ × *C*_*c* GS‐774277_) + (*CLm*_*p* GS‐774277_ × *C*_*p* RDV_) − (*Q*_GS‐774277_ × *C*_*p* GS‐774277_)
$\frac{DA}{DT}$_(GS‐441524 Central compartment)_ = (*CLm*_*c* GS‐441524_ × *C*_*c* GS‐774277_) + (*Q*_GS‐441524_ × *C*_*p* GS‐441524_) − (*CL*_GS‐441524_ × *C*_*c* GS‐441524_) − (*Q*_GS‐441524_ × *C*_*c* GS‐441524_)
$\frac{DA}{DT}$_(GS‐441524 Peripheral compartment)_ = (*Q*_GS‐441524_ × *C*_*c* GS‐441524_) − (*Q*_GS‐441524_ × *C*_*p* GS‐441524_)

Ordinary differential equation pharmacokinetic behavior model was developed based on mean concentration data obtained from phase I clinical trials, where remdesivir was administered in different doses as single-dose 2-h intravenous infusion in healthy subjects. DA/DT represents the change rate of drug amount in the respective compartment. *C*_*c*_ concentration in the central compartment; *C*_*p*_ concentration in the peripheral compartment; *A* the amount of a substance at a time; *CL* total body clearance; *Vd*_*c*_* and Vd*_*p*_ the volume of distribution for central and peripheral compartments, respectively; *Q* inter-compartmental clearance; *CLm* formation clearance of metabolites

As no individual values were available, the obtained variability of fixed parameters reflects inter-cohort variabilities rather than inter-individual variability. And they lump the variability due to differences between volunteers in the different cohorts and they were defined with a log-normal distribution as.

$$\log\left(\theta_i\right)=\log\left(\theta_{\mathrm{population}}\right)+\eta i$$

where *θ*_*i*_ is the estimated parameter for the mean concentration of the *i*th dose, *θ*_population_ is the mean across doses, and *ηi* is a random effect describing the deviation of the PK parameter for the *i*th dose level from the typical PK parameter estimated for all doses. Parameter *ηi* is assumed to follow a normal distribution with a mean of zero and a variance of *ω*^2^.

Additive, proportional, and combined error models were tested:

$$\begin{array}{c}\mathrm{Additive}: {C}_{ij}={Y}_{ij}+\alpha \\ \mathrm{Proportional}: {C}_{ij}={Y}_{ij}+\left(b\right)\times {Y}_{ij}\\ \mathrm{Mixed}: {C}_{ij}={Y}_{ij}+\sqrt{{\left(\alpha \right)}^{2}+{\left(\left(b\right)\times Yij\right)}^{2}}\end{array}$$

Here, *C*_*ij*_ is the observed value for the mean concentration of the dose *i* at time point *j*. *Y*_*ij*_ is the predicted concentration value of the dose *i* at time point *j* estimated by the model. Parameters *a* and *b* are additive and proportional residual errors, respectively.

Simulations were run using R (R Core Team 2021), Simulx 2020R1, and Simulx R package bootstrapping and simulation function (Lixoft, Antony, France), with 256 simulated subjects. To simulate a real-life clinically relevant dosing regimen, the simulation also included a 200 mg, 30-min intravenous infusion of remdesivir on day 1, with subsequent 100 mg, 30-min intravenous infusions for the following 4 days. The choice of this regimen schedule was based on what is recommended by internal hospital physicians, the FDA (U.S. Food and Drug Administration) fact sheet for healthcare providers, and the EMA (European Medicines Agency) Summary of Product Characteristics (CHMP 2020; FDA 2020). The fraction of censored observations for remdesivir, GS‐774277, and GS‐441524 were calculated by determining the ratio of numbers of observations with values below the reported lower limit of quantification to the total number of observations at a time point.

## Results

**Fig. 1:**
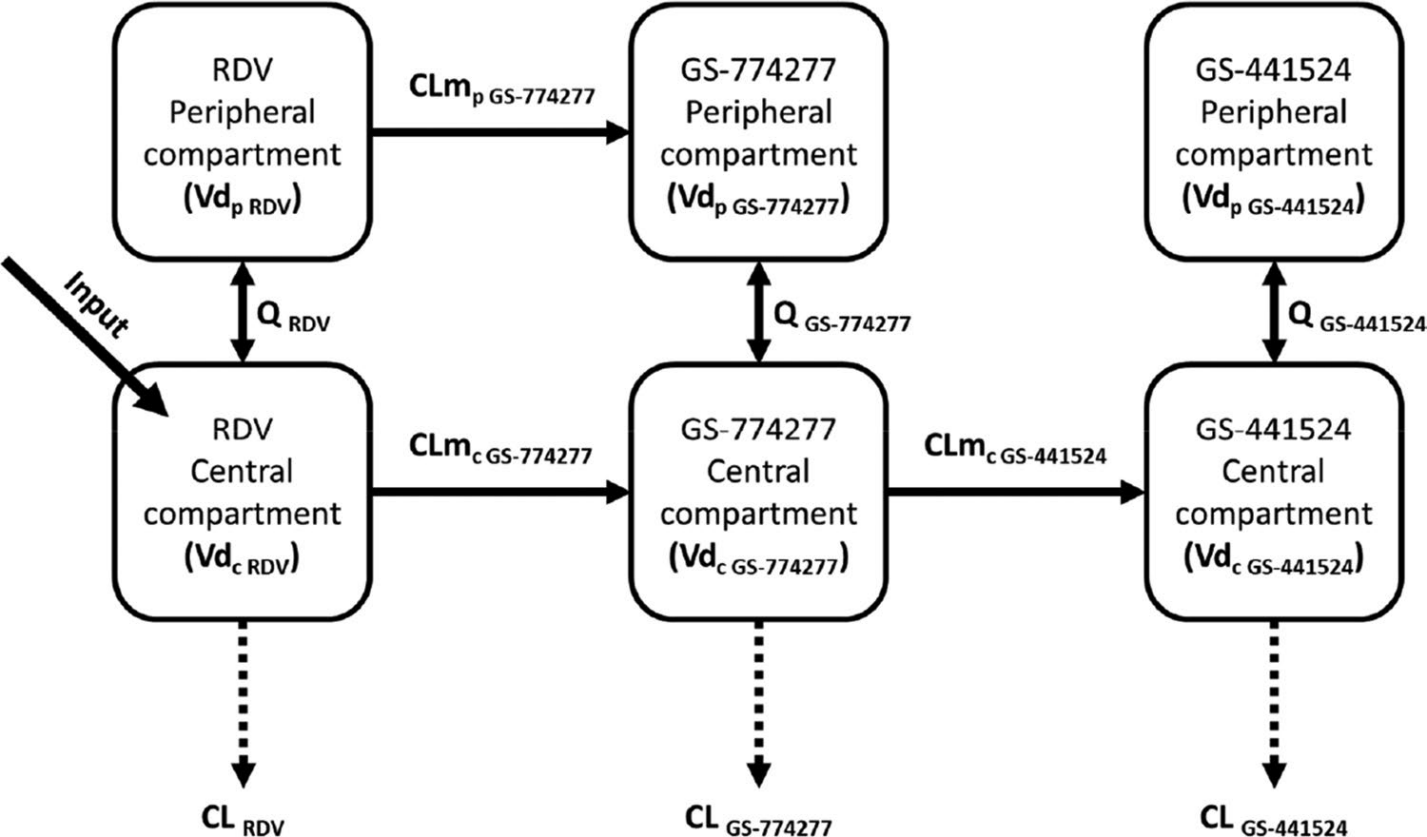
Overview of the final population pharmacokinetic model for remdesivir (RDV) and its metabolites: GS‐704277 and GS‐441524 that was developed using non-linear mixed effect modeling software. The model described each moiety to have a 2-compartment distribution, with sequential metabolism occurring from the central compartment, in addition to remdesivir peripheral metabolism to GS‐774277. And elimination is modeled to occur in the central compartments. *CL* total body clearance (CL), *Vd*_*c*_* and Vd*_*p*_ the volume of distribution for central and peripheral compartments, *Q* inter-compartmental clearance, *CLm* formation clearance of metabolites

The best model describing PK data of all moieties included two compartments for remdesivir and each metabolite. The model suggested metabolism to occur mainly in the central compartment from one moiety to the next one. Additional metabolism was assumed to take place from the peripheral remdesivir compartment to the peripheral GS‐704277 compartment, and elimination was assumed to occur from the central compartments of remdesivir and both metabolites (Fig. 1).

**Fig. 2:**
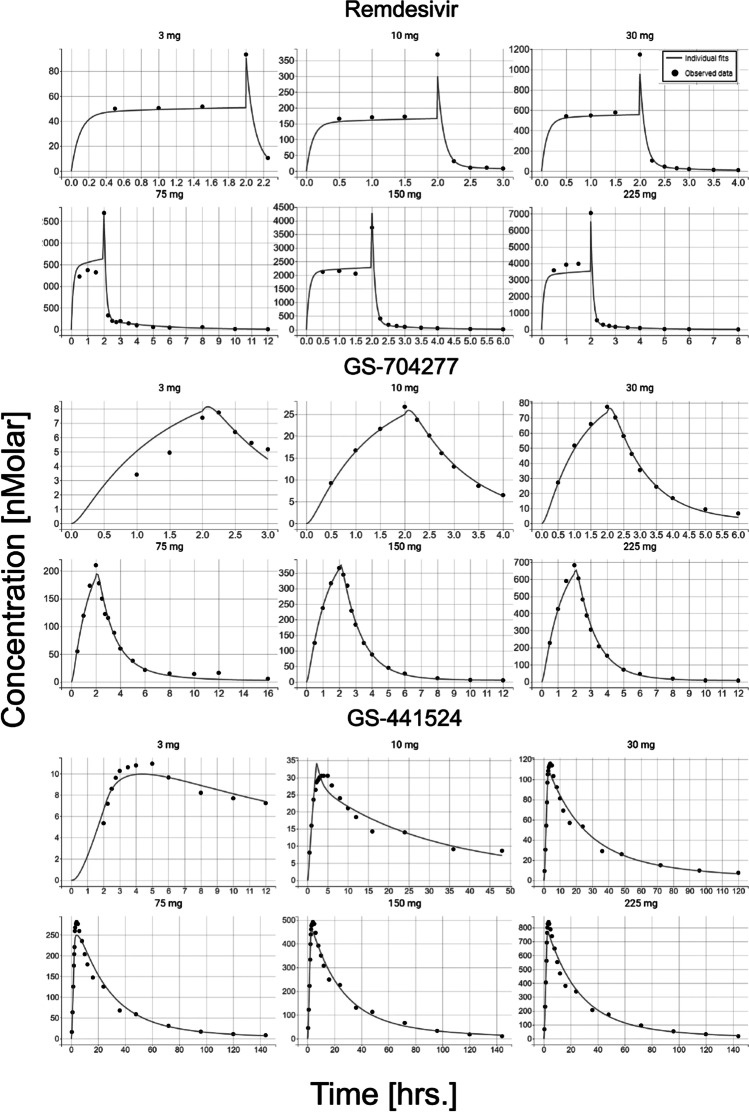
Observed mean concentrations (black dots) and model predictions (black line) of each given dose, following single 2-h intravenous infusion of either 3 mg, 10 mg, 30 mg, 75 mg, 150 mg, or 225 mg of remdesivir

**Fig. 3:**
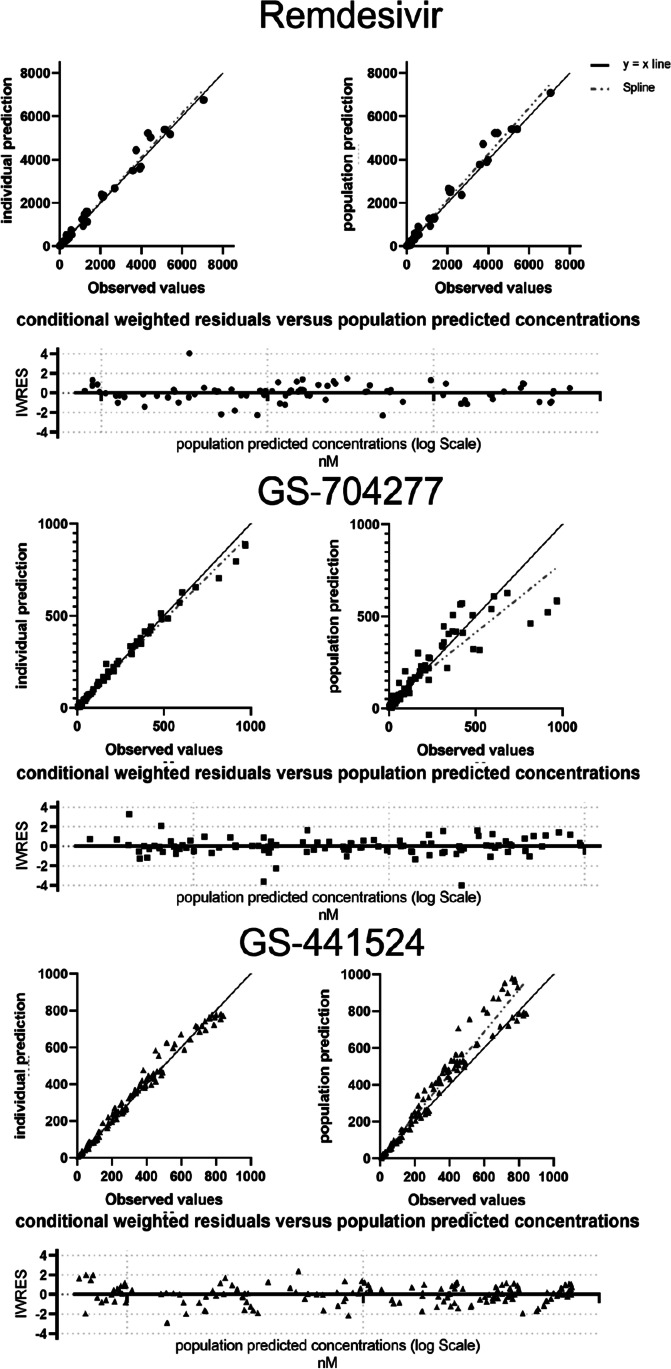
Goodness-of-fit plots describing remdesivir model-predicted plasma concentration value agreement with the observed values

The final model predicted PK profiles for remdesivir, GS‐704277, and GS‐441524 at individual dose levels. The predicted concentration values were in good agreement with the observed concentrations at each time point (Figs. 2 and 3).

Population parameter estimates for the mean of the published plasma concentration‐vs.‐time profiles of remdesivir, GS‐704277, and GS‐441524 following remdesivir 2-h single-dose intravenous administration in healthy volunteers are described in detail in Table 2. Exposure values were similar to previously published remdesivir PK non-compartmental analysis (Table S1). The terminal elimination half‐lives in the central compartment for remdesivir, GS‐704277, and GS‐441524 were 1 h, 1.1 h, and 20 h, respectively.

**Table 2:** Pharmacokinetic population parameter estimates of remdesivir, GS‐441524, and GS‐704277 following remdesivir single-dose administration (2-h infusion) in healthy subjects

PK parameters	Remdesivir	GS‐704277	GS‐441524
Central compartment volume of distribution (L)	4.89	96.4	26.2 (0.71)
Peripheral compartment volume of distribution (L)	46.5	8.64	66.2 (0.24)
Inter-compartmental clearance (L/h)	13.2	0.12	55
Total body clearance (L/h)	18.1 (0.39)	36.9 (0.31)	4.74
Central formation clearance (L/h)	-	16.9 (0.25)	50.5 (0.27)
Peripheral formation clearance (L/h)	-	18.9 (0.53)	-

Population parameter estimates of the fixed effects (SD of the random effects) for remdesivir and its metabolites (GS‐704277 and GS‐441524). The estimates were generated by Monolix software and using mean concentration data points obtained from Gileads’ phase I clinical trials, where remdesivir was administered in doses of 3 mg, 10 mg, 30 mg, 75 mg, 150 mg, and 225 mg as single 2-h intravenous infusion in healthy subjects

The final model had exponential inter-cohort variabilities for the following PK parameters: *Vd*_*c* G GS‐441524_, *Vd*_*p* GS‐441524_, *CL*_RDV_, *CLm*_*p* GS‐774277_, *CLm*_*c* GS‐774277_, *CL*_GS‐774277_, and *CLm*_*c* GS‐441524_ (Table 2). Also, the error models which matched the data best were proportional for remdesivir, combined for GS‐704277, and proportional for GS‐441524.

**Fig. 4:**
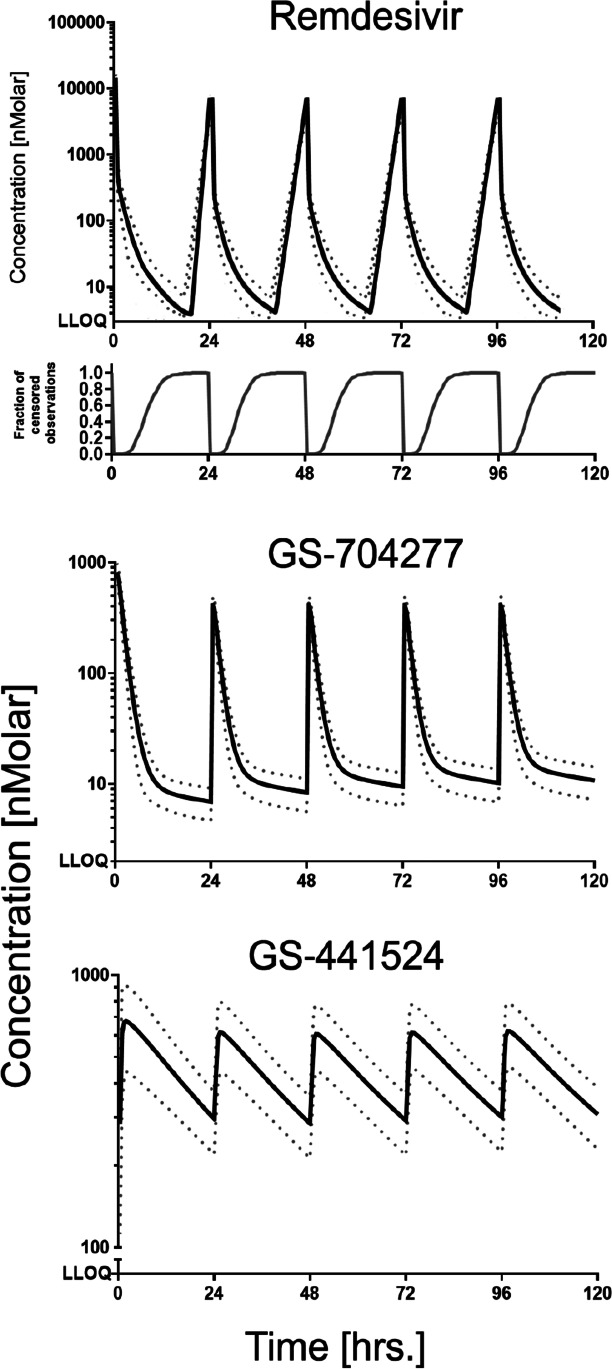
Simulated plasma concentration‐vs.‐time profiles of remdesivir, GS‐704277, and GS‐441524 following a simulated 30-min intravenous infusion of 200 mg of remdesivir on day 1, with 100 mg, 30-min intravenous infusion for the following 4 days. The bottom part of the remdesivir panel shows the fraction of censored observation numbers to the total observations at a given time. There were no censored observations for the metabolites. Dotted lines reflect standard deviation values

We found by simulating remdesivir administration at the recommended dosage that the *C*_max_ (between-dose SD) of remdesivir, GS‐704277, and GS‐441524 were 13.7 µM (2.39), 807 nM (173), and 726 nM (240), respectively, following the initial loading dose. GS‐704277 *t*_max_ was reached immediately after the end of the infusion, while GS‐441524 reached its *t*_max_ 1 h after the end of the infusion. The fraction of censored observations for remdesivir reached the value of 1 after 20 h following the initial 200-mg infusion, and 17 h for each sequential 100-mg infusion. In contrast, GS‐774277 and GS‐441524 did not reach the values of their reported LLOQ during the whole duration of therapy (Fig. 4).

Subsequent *C*_max_ value for GS‐441524, following the administration of multiple 100-mg remdesivir doses, was 645.5 nM (17.57) and the half-life reached 29.36 h both estimated by simulation.

The simulation used the previously generated population parameters of fixed effects, the standard deviation of the random effects, and error model estimates (Figure S3).

## Discussion

In this report, we were able to develop a compartmental non-linear mixed effect model that can describe the mean concentration vs. time course of remdesivir and its two detectable metabolites reasonably well, while also adapting for and describing inter-dose variability.

Both the developed model and the simulations gave similar values for the derived PK parameters (AUC, elimination half-life, *C*_max_) compared to other published non-compartmental analyses of remdesivir, GS‐441524, and GS‐704277 (Tables 2 and S2) (Humeniuk et al. 2020; Tempestilli et al. 2020). The model itself is empirical but physiologically and metabolically plausible. The model incorporates a sequential metabolism from remdesivir to GS‐704277 followed by GS‐704277 metabolism to GS‐441524, which is in agreement with the known metabolic fate of remdesivir (Humeniuk et al. 2021a; Wen et al. 2021). As a peculiarity, the developed model incorporated a peripheral metabolism to GS‐704277, and this does not contradict physiological considerations. A comparison of the parameters to those reported by Sukeishi et al. (2021) is difficult because their evaluation could not consider that a remdesivir dose is not metabolized completely to GS‐441524, and therefore, any values reported indeed are values relative to the fraction metabolized (fm) to GS-441524. Assuming a fraction of about 0.5 (Humeniuk et al. 2021a, b), the basic clearance value reported of about 12 L/h (= CL/fm) corresponds to a true clearance of 6 L/h, which is close to our result of about 5 L/h and also not far from 5.71 L/h value, which was reported by Gilead (Hartman et al. 2020).

We found by the simulation of the clinically approved regimen that plasma concentrations of GS-441524 after 20 min from the beginning of the 200-mg infusion would reach the reported EC_50_ value (180 nM) in SARS-CoV primary human airway epithelial cells (Yan and Muller 2020). The concentration stayed above EC_50_ values throughout the whole simulated 6 days (Fig. 4). Here we assume that lung epithelial cell exposure is close to plasma exposure. However, PBPK modeling predicts that lung concentrations of GS-441524 are several-fold lower than plasma concentration and that GS-441524 plasma concentrations would not be useful to predict lung exposure of the active metabolite GS-443902 (Fan et al. 2021).

At the end, clinical data are required to assess any relationship between plasma pharmacokinetics of remdesivir and its metabolites and efficacy in patients.

A recent study showed that early administration of remdesivir among non-hospitalized patients with at least one risk factor for disease progression could in fact lower the risk of hospitalization or death compared to placebo groups (Gottlieb et al. 2021). However, this study did not associate efficacy with the level of exposure to remdesivir or its metabolite. And so far, the relevance of EC50 level interpretation for remdesivir or its metabolite levels is still unclear.

We attempted to apply our model to the data we reported for a patient with renal impairment (Sörgel et al. 2021) — but we failed. The concentrations in this patient were much higher than those observed by Humeniuk et al. (2020) already at the end of the infusion, which cannot be readily explained by a decreased elimination only. A reason for this could be that the model here is estimating the variability in mean concentrations across cohorts, which might be magnitudes lower than the variability at the level of individual patients.

Thus, further testing by independent datasets including data obtained from various patient populations is required to assess the external validity of this model. Also taking into account that this model was developed from data including only healthy volunteers with a focus on a Hispanic population, and no detailed information regarding age, BMI, or renal and hepatic function was available.

The model lacks variabilities on individual levels and does not consider reported standard errors in the clinical trials, which are considered to be some limitations associated with the model. Further investigations on drug efficacy, target tissues and/or intracellular concentrations, and protein binding are needed for a better understanding of the overall pharmacokinetics of remdesivir. Ideally, a comprehensive population model of remdesivir would also integrate pharmacodynamic data. The present model however may serve as a good starting point for such additional evaluations.

## Supplementary Information

Below is the link to the electronic supplementary material.

Supplementary file1 (DOCX 1760 kb)

## Data Availability

Further data are available from the corresponding author, A. A., upon reasonable request.
