# Supplementary material for: A population pharmacokinetic model of remdesivir and its major metabolites based on published mean values from healthy subjects
Source: Naunyn Schmiedebergs Arch Pharmacol. 2022 Sep 20;396(1):73–82. doi: 10.1007/s00210-022-02292-6 (PMC9485022; doi:10.1007/s00210-022-02292-6)
Supplement: Supplementary file 1 — Supplementary file1 (DOCX 1760 kb) [file 210_2022_2292_MOESM1_ESM.docx]

Metabolic pathway of remdesivir (GS-5734)


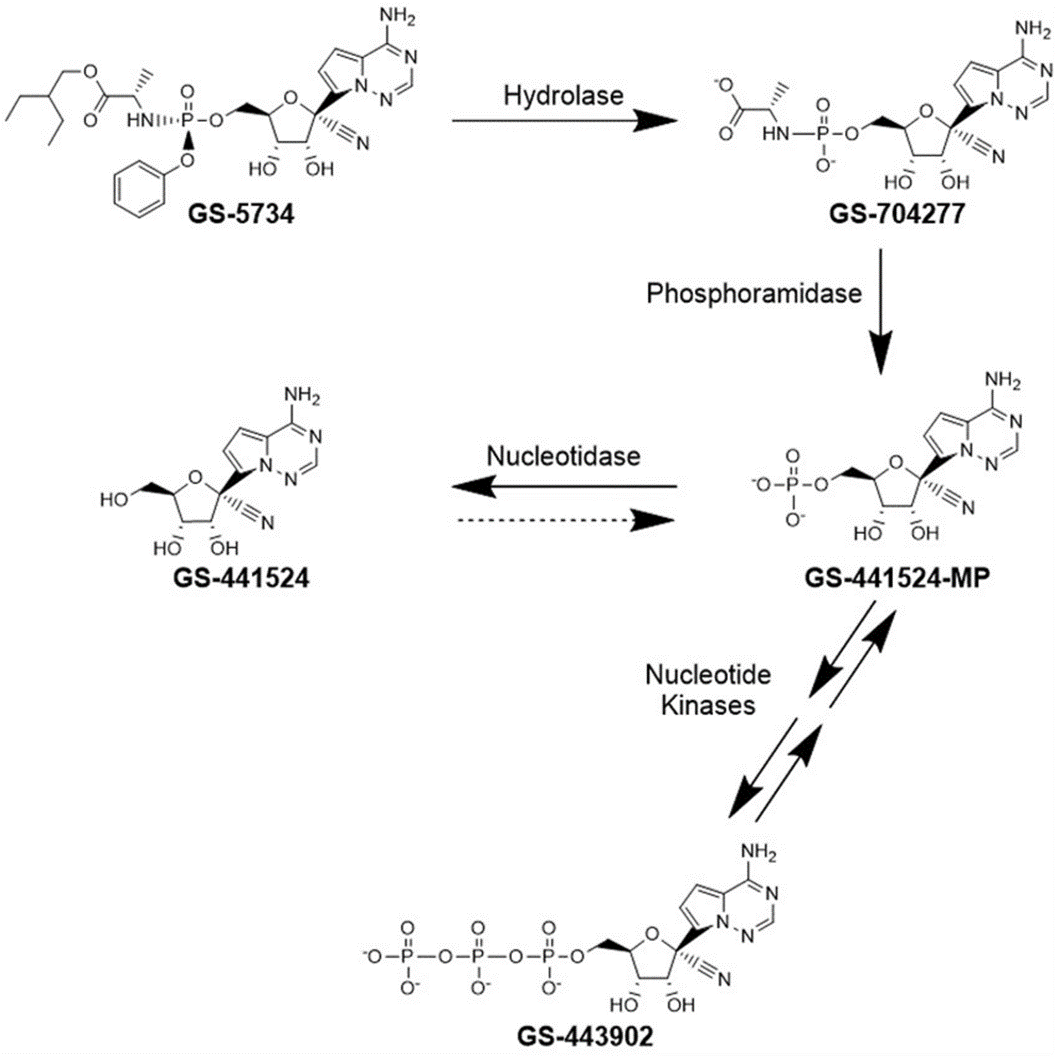


**Figure S1,** Remdesivir undergoes metabolism to form its active moiety, GS-443902. The metabolism process includes cleavage of remdesivir by carboxylesterases to form GS-704277 which can be measured in plasma. Subsequently, metabolism by phosphoramidase results in the formation of GS-441524-monophosphate (MP), which is further phosphorylated to the active nucleoside triphosphate, GS-443902. Dephosphorylation of GS-441524-MP results in the formation of the nucleoside analog, GS-441524 which is the 2^nd^ plasma quantifiable metabolite (Humeniuk et al. 2021b).

-2 x Log of Likelihood estimation of different distribution patterns of Remdesivir, GS‐774277, and GS‐441524 with different metabolic models

**Figure S2,** -2 x Log of Likelihood estimation of different distribution patterns of Remdesivir, GS‐774277, and GS‐441524 with different metabolic models that control the conversion of Remdesivir to GS‐704277, and GS‐704277 to GS‐441524 following the conversion of the models using Monolix

Distribution of Model parameters used for the simulation of remdesivir, GS‐704277, and GS‐441524 exposure following administration of the clinically used regimen

**Figure S3** Fixed model parameters of remdesivir (RDV), GS‐704277, and GS‐441524 following a simulated 30-minute 200 mg intravenous infusion of remdesivir on day one, with 100 mg, 30 minutes intravenous infusion for the following 4 days. Total body clearance (CL); the volume of distribution for different compartments (Vdc, and Vdp), intercompartmental clearance (Q); formation clearance of metabolites (CLm). Dotted values resemble outliers according to Tukey's range test.

Simulation of plasma concentration‐vs‐time profiles for remdesivir, GS‐704277, and GS‐441524 following 2 hours of Intravenous infusion of either 3 mg, 10 mg, 30 mg, 75 mg, 150 mg, or 225 mg of remdesivir

**Figure S4** Doses of 3 mg, 10 mg, 30mg, 75mg, 150mg, and 225 mg of remdesivir were simulated as a single two hours infusion followed by a saline flush using previously obtained population parameter estimates. Simulation of remdesivir dosing was run as a 2 hours infusion using the bootstrapping function in Simulx package in R,.
